# The integration of social and neural synchrony: a case for ecologically valid research using MEG neuroimaging

**DOI:** 10.1093/scan/nsaa061

**Published:** 2020-05-07

**Authors:** Jonathan Levy, Kaisu Lankinen, Maria Hakonen, Ruth Feldman

**Affiliations:** Department of Neuroscience and Biomedical Engineering, Aalto University, 02150 Espoo, Finland; Interdisciplinary Center, Baruch Ivcher School of Psychology, Herzliya 46150, Israel; Department of Radiology, Athinoula A. Martinos Center for Biomedical Imaging, Massachusetts General Hospital, Charlestown, MA, USA; Department of Radiology, Harvard Medical School, Boston, MA, USA; Department of Neuroscience and Biomedical Engineering, Aalto University, 02150 Espoo, Finland; Faculty of Sport and Health Sciences, University of Jyväskylä, Jyväskylä, Finland; Interdisciplinary Center, Baruch Ivcher School of Psychology, Herzliya 46150, Israel; Yale University, Child Study Center, New Haven, CT 06520, USA

**Keywords:** neural synchrony, social interaction, MEG, ecological validity, social neuroscience

## Abstract

The recent decade has seen a shift from artificial and environmentally deprived experiments in neuroscience to real-life studies on multiple brains in interaction, coordination and synchrony. In these new interpersonal synchrony experiments, there has been a growing trend to employ naturalistic social interactions to evaluate mechanisms underlying synchronous neuronal communication. Here, we emphasize the importance of integrating the assessment of neural synchrony with measurement of nonverbal behavioral synchrony as expressed in various social contexts: relaxed social interactions, planning a joint pleasurable activity, conflict discussion, invocation of trauma, or support giving and assess the integration of neural and behavioral synchrony across developmental stages and psychopathological conditions. We also showcase the advantages of magnetoencephalography neuroimaging as a promising tool for studying interactive neural synchrony and consider the challenge of ecological validity at the expense of experimental rigor. We review recent evidence of rhythmic information flow between brains in interaction and conclude with addressing state-of-the-art developments that may contribute to advance research on brain-to-brain coordination to the next level.

## Toward a real-life interactive neuroscience

A fundamental feature of human life is the ‘hypersociality’ of our species, the innate need and exquisite capacity for social collaboration, group living, affiliation and cooperation that have led *Homo sapiens* to coordinate action toward a joint goal and construct complicated communicative signal systems, which, according to some authors, are the main reasons for the survival and thriving of our species (Dunbar, 1998; Wilson, 2012). A central mechanism underpinning human sociality is biobehavioral synchrony, the human capacity to coordinate physiological processes between interactive partners during moments of social contact, including the coordination of heart rhythms, hormonal release, neural oscillations and brain activations (Feldman, 2012, 2016, 2017; Levy *et al.*, 2017). A key tenet of the biobehavioral synchrony model is that physiological coordination is triggered in a bottom-up way and depends on the coordination of social action, such as motor activity, facial mimicking or the synchrony of nonverbal interactive signals, including shared gaze, joint laugh or mutual expression of positive affect and that such behavioral synchrony provides the template for the synchrony of neural processes (Feldman, 2017). The model maintains that the synchrony of neural and behavioral process is a key feature of the mother–infant bonding context in mammals, where the mature maternal brain externally regulates the infant’s immature brain and tunes it to social living. These early attachment experiences are then transferred to other social affiliations throughout life, such as romantic relationships or close friendships, and both animal (Numan and Young, 2016) and human studies (Feldman, 2016) have shown that parental and pair bonding utilize similar neural, endocrine, molecular and behavioral processes. These early attachment experiences also prepare the brain to life within social groups and enable the individual to synchronize both physiology and social action with unfamiliar strangers. As such, the parent–child attachment context, as well as other affiliative bonds, may serve as a good vantage point for research on human sociality, particularly the integration of social and neural synchrony during moments of social contact. Such research requires new, ecologically valid paradigms to capture the richness of human social life within its natural ecology. In the following section, we address the study of interpersonal neural synchrony. We outline the progress from single person to multiperson neuroscience, the technological advances that afforded this transition, and the methodological challenges that phase this new area of research.

Over the past five years there has been an exponential boom in neuroscientific studies of interpersonal social interaction (Redcay and Schilbach, 2019). Initially, to unveil the underlying mechanism of social interaction, studies investigated the neural correlates related to artificial social stimuli. Although these studies applied decontextualized experimental environments, there has been a steady move from well-controlled socially deprived experiments to naturalistic and socially dynamic stimuli (Hari *et al.*, 2015). Yet, even such dynamic stimuli were studied in the context of isolated brains, thereby neglecting the valuable information of the dynamic interaction among multiple brains. This aspect was particularly missing because in real life, human societies rely on complex and large-scale social interactions among multiple individuals. During our daily life we constantly receive information from social partners and must simultaneously respond, update predictions and monitor actions in dynamic and multifaceted ways according to person, place and the task at hand.

**Fig. 1:**
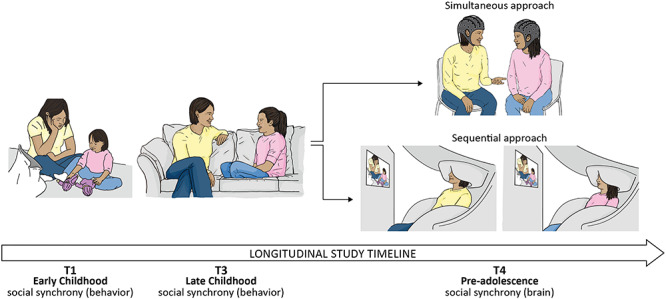
The integration of socio-behavioral and neural synchrony approach. An example of a longitudinal study integrating socio-behavioral interaction (left and middle panels) together with paradigms measuring interbrain synchrony: the simultaneous approach (right upper panel) favors ecological validity whereas a sequential approach in magnetoencephalography (right upper panel) achieves a balance between ecological validity and experimental rigor.

In parallel, recent technological developments in portable devices enabled a paradigm shift toward a nonreductionist, multibrain approach to social interactions. Hyperscanning, the simultaneous data collection from two or more brains (Figure 1, upper right panel), was the term originally coined to multibrain neuroimaging, and since its first appearance (Montague *et al.*, 2002) there has been a steady rise in the number of studies set to investigate the neural mechanisms that underpin the interactions among multiple brains in a variety of social tasks and interactive settings. The early hyperscanning studies typically addressed slow-paced social interactions, such as movement imitation of hand movement (Dumas *et al.*, 2010). These studies targeted real social interactions, but tended to tap reactive, rather than interactive processes (Hari *et al.*, 2015). Yet, owing to recent advances in neuroimaging technologies, we are currently witnessing a surge in studies that tackle the real-life dynamics of social interactions and employ quick and naturalistic experimental designs (Shamay-tsoory and Mendelsohn, 2019). A major emphasis in this paradigm shift was the ecological validity of these studies that aimed to capture how brains function ‘in the wild’ (Sonkusare *et al.*, 2019). For instance, experiments tested dyadic social conversation (Kinreich *et al.*, 2017), humming (Osaka *et al.*, 2014), guitar playing (Müller *et al.*, 2013), empathic touch (Goldstein *et al.*, 2018) or classroom student learning (Dikker *et al.*, 2017). Overall, these studies demonstrated that during coordinated joint actions there is enhanced neural synchrony, confirming the prediction of our biobehavioral model on the bottom-up, behavior-based nature of brain-to-brain synchrony (Feldman, 2016, 2017).

Despite the temptation to ascribe the putative mechanism of neural synchrony to various interpersonal interactions, it is important to reflect upon the phenomenon of neural synchrony. It is still unclear whether neural synchrony always implies interpersonal communication, that is, dyadic (or intragroup) neural information flow among individuals. According to Burgess, synchrony does not necessarily imply reciprocal information exchange (Burgess, 2013). Rather, the synchrony of any physical system may, for instance, be induced by a common external driver, such as the perception of an identical external stimulus and in such cases, synchronization may occur despite the lack of interpersonal communication. Alternatively, synchrony may be driven by one of the agents in the dyad but without reciprocity. Another type of synchrony may occur by coincidence; for example, between two brains that are in separate physical locations, both operating in a similar frequency band and it is likely that these two brains will operate synchronously for at least a limited period of time. This means that given the intrinsic properties of the human brain, neural synchrony can be observed despite the lack of any interbrain communication. Moreover, spurious measures of neural synchrony can also result from cardiac or respiratory synchrony (Müller and Lindenberger, 2011), thereby misleading interpretations of the data. As such, it is important not only to apply great caution in the interpretation of interbrain findings but also to complement research on neural coordination with careful observations of behavioral synchrony.

The application of specific analysis schemes, for example, circular correlation (Burgess, 2013) is thought to be more sensitive to the detection of reciprocal information exchange, and less prone to measuring spurious correlations. This taps into another issue related to the evaluation of neural synchrony. To date, most approaches rely on correlation metrics originally developed for within-brain connectivity analyses, for instance, coherence or phase synchrony (Lachaux *et al.*, 1999). However, real-life interactions involve more complex dependencies between brain signals, such as variance in signal time-lags and varying directionality between the dyadic signals (Hari *et al.*, 2015). Hence, nonlinear correlations (Campi *et al.*, 2013), multivariate methods (as will be elaborated later in this article) and other computational alternatives are important to develop and implement for improving the accuracy of estimating neural synchrony.

In light of these considerations, it is crucial to be cautious when observing neural synchrony and to take specific experimental and analytical measures in order to avoid erroneous interpretation of hyperscanning data. In addition to the analytical improvements mentioned above, several other recommendations may help against misinterpretation of neural synchrony data, including the use of several experimental controls and reference measures (Goldstein *et al.*, 2018), the combination of naturalistic and controlled settings (Leong *et al.*, 2017) and the integration of neural synchrony with rich and real-life indices of synchronous behavior (Kinreich *et al.*, 2017). In the next two sections, we address the last two points, respectively. Although capturing true neural synchrony remains a grand challenge in the field, it is recommended that several other methodological steps, neuroimaging approaches (e.g. magnetoencephalography) and conceptual discussions should be implemented by researchers to improve the chances of reporting true neural synchrony in interpersonal studies.

## The trade-off between ecological validity and experimental control

Another aspect in hyperscanning studies that requires careful consideration is the trade-off between ecological validity and experimental rigor. Such risk may be intrinsically embedded in the framework of ‘ecological validity’, notwithstanding the great potential that ecologically valid experiments hold. First, social interaction is a multifaceted phenomenon, which is difficult to be distilled as a single process, and hence may be process-unspecific (Wheatley *et al.*, 2019). Second, under natural interactional settings, humans perform various forms of bodily movements that are accompanied not only by sensorimotor activity, but also by non-neural noisy activity. Obviously, our stand is that research should not abandon real-life hyperscanning for the sake of experimental control. We certainly need to study real-life social interactions while bearing in mind that the natural context of this approach is multimodal, interactive and not as well-controlled or specific. For instance, multimodality is enhanced during social interactions because of the different behavioral and expression channels (e.g. body, face and eyes), by which interaction is conveyed (Wheatley *et al.*, 2019). Similarly, the interactive context should be taken into account given the fast, moment-by-moment variability occurring during social interaction (Levy *et al.*, 2017); hence, applying a time-resolved approach while coding and analyzing social interactions is needed, which, while enriching our understanding the neural basis of social interactions adds to the complexity of interpreting real-life interactional data.

This new challenge to find an efficient trade-off between ecological validity and experimental rigor pushes social neuroscientists to propose creative approaches. One approach that respects the need for such balance is sequential dual-brain imaging (Schippers *et al.*, 2010), which allows to artificially study information transfer between social partners. These studies often image the two individuals in separate scanning sessions, thereby simulating social interaction. For instance, in a recent study, Leong *et al.* (2017) investigated neural communication between adults and infants during moments of social gaze. They proposed an inventive way to do so by first imaging the adult brain singing (and videotaping this session) and then showing the video of the adult singing to the infants while imaging their brains. Such sequential settings are tightly controlled and allow to mechanistically test cognitive processes underlying social interaction. Nevertheless, sequential dual-brain scans offer an artificial simulation of the actual neural mechanisms underlying social interaction. We support the methodological approach advocating that good science should combine both sequential (i.e. rigorous approach) and simultaneous (i.e. realistic approach) brain scanning (Cantlon, 2020) to offer valuable insight into the dual working of brains in interaction. Yet, implementing both controlled and real-life settings in a single study is not always possible; in contrast to semipassive interactions (e.g. gaze and rhyming) (Leong *et al.*, 2017), it is difficult to implement controlled, sequential scanning during active social interactions. In the second part of this manuscript, we elaborate on the use of magnetoencephalography (MEG) for obtaining a good trade-off between ecological validity and experimental control.

## Integrating behavioral and neural synchrony

To investigate neural synchrony, most studies relied on contrasting two experimental conditions/groups (Hu *et al.*, 2017; Pérez *et al.*, 2017; Goldstein *et al.*, 2018). For instance, Hu *et al.* (2017) studied interbrain synchrony by contrasting two experimental groups: participants who performed a coordination task *vs* participants who performed an independence task. In more recent studies, however, the coding of nonverbal social behavior was incorporated to supplement and fine-tune the mechanisms of neural synchrony, consistent with the theoretical propositions of the biobehavioral synchrony model proposing that social action provides a template for neural coordination. For example, body-movement synchrony (Galbusera *et al.*, 2019) was behaviorally coded to study emotion regulation, or tapping/drumming synchrony was coded and correlated to neural synchrony (Dai *et al.*, 2018; Rojiani *et al.*, 2018). To better understand the neural determinants of social communication via eye-to-eye contact, Hirsch *et al.* (2017) used eye-tracking and functional near-infrared spectroscopy (fNIRS) neuroimaging. They analyzed neural coordination during joint eye-gaze, and inspected within- and across-brain neural correlates of eye-to-eye contact; they found a network that mediates neural responses during eye-to-eye contact between dyads. Similarly, Leong *et al.* (2017) manipulated gaze and coded participants’ vocalizations during gaze episodes. They found that direct gaze strengthens interpersonal neural communication—a pattern replicated in two experiments: one more controlled and the other more ecologically valid. To measure the influence of attention on neural synchrony, Wass *et al.* (2018) manually coded visual attentional patterns of parents and infants by reviewing their respective video recordings on a frame-by-frame basis. The authors found that parents’ neural oscillatory power closely tracked and responded to changes in their infants’ attention. Moreover, several studies tested higher order social processes, such as cooperation and attachment. Numerous studies set to understand the association between neural synchrony and overall behavioral cooperation, collected third-person rating (Lu and Hao, 2019) or computed scores from computer tasks (Mu *et al.*, 2017; Reindl *et al.*, 2018; Miller *et al.*, 2019) and proposed neural determinants of interpersonal behavioral cooperation. Authors also measured the perception of attachment cues, and found that it allocates considerable neural resources by eliciting a strong neural oscillatory response and the strength of the neural response was associated with the degree of reciprocity in the attachment relationship (Pratt *et al.*, 2018). Others compared different attachment styles during interactions to relate it to patterns of neural synchrony (Miller *et al.*, 2019).

It is important to note, however, that attempting to locate a common neural denominator underlying interactional hyperscanning studies remains challenging at this stage. Several studies reported the recruitment of the temporal cortex (Hirsch *et al.*, 2017; Kinreich *et al.*, 2017; Rojiani *et al.*, 2018); however, it appears that many more studies reported that interpersonal neural synchrony recruits the prefrontal cortex (Babiloni *et al.*, 2012; Konvalinka *et al.*, 2014; Hirsch *et al.*, 2017; Hu *et al.*, 2017; Dai *et al.*, 2018; Reindl *et al.*, 2018; Lu and Hao, 2019; Miller *et al.*, 2019; Piazza *et al.*, 2020). Likewise, oscillatory activity has been quite multirhythmic, although gamma (Kinreich *et al.*, 2017; Levy *et al.*, 2017; Mu *et al.*, 2017; Pratt *et al.*, 2018) and particularly alpha (Dumas *et al.*, 2010; Babiloni *et al.*, 2012; Kawasaki *et al.*, 2013; Konvalinka *et al.*, 2014; Leong *et al.*, 2017; Pérez *et al.*, 2017; Goldstein *et al.*, 2018; Pratt *et al.*, 2018; Wass *et al.*, 2018) rhythms have been reported to synchronize in these hyperscanning studies. Overall, it appears that several neural mechanisms underlie interpersonal neural synchrony, and common findings pinpoint alpha activity in prefrontal regions, alpha activity in central regions and gamma activity in temporal regions (Kinreich *et al.*, 2017). Yet, the richness and heterogeneity of current experimental designs, methodology and scope of research render it premature to infer a common neural network and describe neural mechanisms that underpin interpersonal neural synchrony. Yet, this line of work advances insight and progress toward a clear definition of the elemental neural mechanisms underlying various modes of interpersonal expression and communication.

Our own work, based on the biobehavioral synchrony model, proposes that neural synchrony is triggered by nonverbal behavioral synchrony and that neural and behavioral synchrony are mutually influencing and their interplay follows two types of mechanisms: enhancement and complementarity (Djalovski *et al.*, 2020, submitted for publication). We further suggest that the integration of social and neural synchrony may be differentially expressed in various social contexts, whether these are multiple one-on-one contexts, such as relaxed social interaction, conflict, planning a joint pleasurable activity, or when partners provide empathic support to each other’s daily hassles, or larger group contexts when groups are involved in various joint activities. In addition, we postulate that the interplay of behavioral and neural synchrony depends on the nature of the relationship between the interacting partners, for instance, among couples, close friends, parent–child dyads, acquaintances or complete strangers. Similarly, in studies of intergroup neural synchrony, the degree of neural coordination would vary according to the amount of training for motor/social synchrony, for instance, in combat units or sports teams, on the degree of familiarity, e.g. classroom students, or, in the case of strangers, on whether or not the group shares a common cause (e.g. a political rally) (Feldman, 2020) In a study testing the impact of combat training on social perception, we coded behavioral synchrony of social vignettes involving group activity, for instance, an army unit in joint synchronous activity or a group of friends socially drinking together around a table, and measured the neural oscillatory response each stimuli evoked in two groups; war veterans who have been in a life-threatening battle and nonveterans controls. Participants were imaged twice, once after intranasal administration of oxytocin and once under placebo. We found that salient experiences in social groups (i.e. combat veterans) shape social perception and the neural oscillations that underlie it: oxytocin, a central social neuropeptide, selectively modulated brain response to social synchrony in the mirror-neurons network (pSTS, IPL and IFG). The two functions of oxytocin, that is, as enhancer or as anxiolytic, were highly dependent on salient experiences within social groups (Levy, Goldstein, Zagoory-Sharon, *et al.*, 2016b). This study further strengthens the proposal that synchrony is a complex phenomenon involving behavioral, social, hormonal and neural determinants, particularly alpha rhythmic activity in the frontal and temporal cortices consistent with some of the aforementioned studies.

Using a longitudinal approach (Figure 1) for integrating behavioral and neural indices of synchrony and for understanding the brain basis of empathy is a new strategy that we recently implemented in several studies. In one such study, we reported that indices of behavioral synchrony and emotions drive future synchrony between two brains during social interactions (Levy *et al.*, 2017) in gamma rhythmic activity in the temporal cortex, consistent with some of the aforementioned research. In another longitudinal study, mother–child behavioral synchrony was monitored throughout childhood. A decade late, we assessed the neural empathic response and found that it was predicted by the degree of behavioral synchrony between mother and child experienced across the first decade of life (Levy, Yirmiya, *et al.*, 2019b; Levy, Goldstein, *et al.*, 2019a). Likewise, we found that trauma exposure reduced connectivity of the default mode network in mother and child, impacting theta connectivity in children and alpha connectivity in mothers, and that the degree of disruption to default mode network connectivity was longitudinally linked to reduced behavioral synchrony across the first decade (Zeev-Wolf *et al.*, 2018). Processes of neural and behavioral synchrony are not solely confined to dyadic settings. In another study on the impact of intergroup conflict on neural synchrony, we investigated neural synchrony within an ethnic group of Jewish–Israeli adolescents, and within an ethnic group of Arab–Palestinian adolescents. These adolescents participated in an MEG experiment that probed the neural empathic response to ingroup and outgroup targets. We found that nonverbal empathic behavior strongly correlated with neural synchrony within one ethnic group (i.e. Arab–Palestinians), but not with another (i.e. Jewish–Israeli) (Levy, Goldstein, Influs, *et al.*, 2016a). This ethnic driving influence was suggested to stem from ethnocentricity and minority mentality of the first group. In another intergroup conflict study, it was found that exogenous oxytocin administration enhanced within-group behavioral coordination (Zhang *et al.*, 2019). In an fNIRS study, authors measured neural synchrony in groups of three and found that synchrony was enhanced between leaders and followers as compared to among followers, thereby suggesting a neural synchronous mechanism underpinning communication of leaders (Jiang *et al.*, 2015). Another group study implementing naturalistic hyperscanning electroencephalographic (EEG) recording inside the classroom (Dikker *et al.*, 2017) found a direct link between neural group synchrony and social behavior (e.g. engagement and dynamics).

Studying neural and behavioral synchrony within-group settings can therefore investigate more complex social dynamics and processes as shown here, for instance, intergroup conflicts, group emotions, leader-group relations or group learning. Despite these interesting aspects, to date there have been only few studies integrating neural and behavioral synchrony within-group settings, perhaps due to the complexity of signal interpretation, but also possibly stemming from the ‘*many minds problem*’, which is particularly prominent during group conversation (Cooney *et al.*, 2020). It therefore remains a methodological challenge to complement the shift from single- to two-person neuroscience, and to further advance to group neuroscience. Altogether, accumulating data suggest that the integration of behavior with neural synchrony is promising for elucidating social phenomena. Altogether these studies advance our understanding of behavioral and neural synchrony in complex dyadic or group settings, for instance, by informing political decision makers, intergroup intervention builders or education policies. These studies also have important implications to psychopathology and high-risk populations, by proposing recommendations for psychological and psychiatric venues of clinical therapy and social support for dyadic relationship and child caregiving.

In several studies we examined using MEG the neural oscillatory response in children while being exposed to videos of their own mother–child interactions compared to unfamiliar mother–child pairs. We used interaction from the children’s early childhood filmed in their home environment for ecological validity and to invoke a keener sense of attachment reminders. In the first study, we found that own mother–child interaction elicited a widespread neural response across temporal and insular cortices, including the insula, STS/STG, and Fusiform Gyrus, as well as the visual cortex. Furthermore, response to attachment cues integrated multiple rhythms, including alpha, beta and gamma, underscoring the significant neural resources the brain allocates to attachment cues, which are so critical for survival (Pratt *et al.*, 2018). We have previously found in a study of children, adolescents and adults, that the integration of multiple oscillatory rhythms in the social brain is a marker of maturity (Levy *et al.*, 2018), and, thus, the multirhythmic response to attachment cues suggest that attachment stimuli elicit and integrated response that fosters the maturation of the social brain. It is noteworthy that these studies, which indirectly involve interactional neural synchrony, also report alpha and gamma rhythmic activity in the temporal cortex, similarly to the hyperscanning studies reviewed above.

Finally, in a similar study that exposed preadolescents who were reared by chronically depressed mothers across the first years of life, *vs* healthy controls, we found that children of depressed mothers, particularly those who later developed affective disorders, showed diminished neural response in beta and gamma rhythms to attachment cues and no differentiation between neural response to own *vs* unfamiliar interaction, indicating no neural exclusivity of the attachment target. For all children, the degree of gamma responsivity to attachment cues was predicted by the level of mother–child behavioral synchrony in early childhood, further supporting the role of synchronous interactions in tuning the brain to social stimuli (Pratt *et al.*, 2019), and in line with the gamma rhythmic activity in the temporal cortex as reviewed above in several hyperscanning studies. Several ongoing studies in our lab use hyperscanning EEG to examine the integration of behavioral and neural synchrony online during social interactions between affiliated partners, such as mothers and children, mothers and adolescents, romantic couples and close friends.

In one study, we measured synchrony between romantic partners in a stable relationship, compared to a male–female stranger couples, during a novel and positive social task, planning a fun day to spend together that will be ‘the best day ever’. Romantic couples showed greater synchrony compared to strangers and the difference was observed in gamma rhythms in temporal regions, consistent with the MEG findings between mothers and children and highlighting temporal gamma as a potential mechanism of brain-to-brain synchrony. Furthermore, we found that moments of neural synchrony were anchored in episodes of behavioral synchrony; when partners synchronized their gaze or positive affect, neural synchrony was observed, but synchrony was not significant above baseline when partners did not coordinate social behavior (Kinreich *et al.*, 2017). These findings provide evidence to our hypothesis indicating that moments of nonverbal synchrony, which are engraved in the brain during early sensitive periods within attachment contexts, function as the foundation upon which individuals can coordinate their brain response online during social interactions within other affiliations (Feldman, 2015). We will examine in the upcoming section how this approach can be efficiently implemented using MEG.

## MEG technology as a promising tool for studying interactional neural synchrony

One neuroimaging device that may mitigate the trade-off between ecological validity and experimental control is MEG. MEG records the magnetic fields generated by the electrical activity of neuronal populations. A key advantage of MEG over other techniques is that it can record brain activity directly and noninvasively, while bypassing the problems commonly caused by intermediate processes: neurovascular coupling in functional magnetic resonance imaging (fMRI) or fNIRS, and scalp-based signal distortion in EEG (Baillet, 2017; Gross, 2019). Aside the general uniqueness of MEG as a neuroimaging tool, we contend that it involves special utility in interactive neural synchrony studies for two main reasons: (i) employing indirect approaches while maintaining controlled settings and (ii) temporal precision and rhythmic activity sustaining social interaction. We will expand on these points in this subsection.

There are few MEG systems in the world, making it challenging to conduct hyperscanning MEG. One of the only studies that enabled such endeavor during dyadic interaction was conducted in Japan (Hirata *et al.*, 2014), while other studies achieved hyperscanning between two cross-site MEG systems (Baess *et al.*, 2012; Remes *et al.*, 2013; Zhdanov *et al.*, 2015). Notwithstanding the innovation, rich neural information and experimental rigor applied in these pioneering studies, they were quite poor in ecological validity. In fact, this brings up an inherent, current problem in most existing MEG systems participants are constrained to remain physically immobile in a magnetically shielded room, thus poorly emulating real-life interaction. Is there a better way to exploit the unique neural information recorded in MEG during social interaction, while at the same time, probing real-life interaction?

Recently we highlighted an approach that attempted at such integration (Levy *et al.*, 2017) by studying the perception of social interaction (i.e. synchrony) in sequential dual-brain settings (Figure 1, bottom right panel). This approach has been motivated by the pioneering fMRI studies that investigated neural intersubject correlation (Hasson *et al.*, 2004), which is a measure of neural synchrony while perceiving identical ongoing stimuli, such as video vignettes, without any communication nor interaction among viewers. Another sequential strategy that does include interaction involves the imaging of one brain transmitting a message (i.e. information) during a prior scanning session, and then exposing the other partner to that message in a subsequent imaging session (Schippers *et al.*, 2010). One advantage of this interactive yet sequential approach is that causality can be estimated within the dyad, under quite controlled settings, thereby increasing signal to noise ratio. Although the noninteractive sequential approach does not involve communication, and therefore is far from emulating real-life social interaction, we proposed an interesting strategy because this sequential measure does yield shared information processing, the information to be processed can be a vignette of dyadic synchronous interaction (Levy *et al.*, 2017). Prior neuroimaging studies demonstrated overlapping neural circuits underpinning the perception and experience of social functions (Singer *et al.*, 2004; Mukamel *et al.*, 2010). Further, examining independent lines of recent research may be taken as indication that the perception of social interaction relies on similar neural substrates compared to social interaction substrates; it is surprising that both studies that tested perception of interaction (Levy, Goldstein, Zagoory-Sharon, *et al.*, 2016b; Isik *et al.*, 2017; Pratt *et al.*, 2018; Walbrin *et al.*, 2018; Arioli and Canessa, 2019), and interaction per se (Kinreich *et al.*, 2017; Sliwa and Freiwald, 2017) highlighted the selective function of the STS in underlying these processes. Hence, as real-time hyperscanning of interacting individuals under ecologically valid conditions is currently methodologically challenging (Hari *et al.*, 2015), this new strategy of well-controlled experimental perception of real-life social interaction may afford an indirect, yet useful vantage point on brain-to-brain coordination using MEG.

Another aspect that MEG can potentially unveil is the rich dynamic information flow between brains in interaction. MEG confers the ability to separate simultaneous active brain regions and the activity dynamics taking place in there. Furthermore, this information can actually be transformed to representations in the frequency domain, thereby illustrating neural generators of multiple rhythms and oscillations—an aspect of brain activity that has been thus far neglected social neuroscience (Stanley and Adolphs, 2013). Neural oscillations are a pervasive feature of neuronal activity in the cerebral cortex, and gamma-band oscillations have been suggested to underlie neural communication (Fries, 2005) and therefore might be important to explore during social interactions. Unfortunately, except MEG, noninvasive methods cannot reliably record gamma-band activity because of signal distortion and the high involvement of this rhythm in underlying movements. This advantage of MEG in recording gamma-band activity is therefore very advantageous for studying social interactions, and accumulating evidence points to the involvement of the gamma rhythm in the perception of social interaction (Levy *et al.*, 2017; Pratt *et al.*, 2018) as well as in social interaction (Kinreich *et al.*, 2017; Mu *et al.*, 2017). For instance, in our recent studies on dyadic social interaction, we found that behavioral synchrony generated interbrain synchrony in the gamma-band, and this synchrony appeared to be generated by the temporal cortex (STS). The validity of this result pattern was strengthened as it was observed both during online interaction (Kinreich *et al.*, 2017) as well as under controlled settings of sequential scans (Levy *et al.*, 2017). Finally, recent concepts relate oscillations at different frequencies to the routing of information flow in the brain and the signaling of predictions and prediction errors (Bastos *et al.*, 2012; Ploner *et al.*, 2016). As social interaction involves rapid and complex mental representations requiring constant predictions and updating prior expectations, studying interactive neural synchrony should be informed by oscillatory information in our brains. In our recent MEG studies we showed that such multirhythmic representations might indeed convey predictions and updates during empathic (Levy *et al.*, 2018) and attachment (Pratt *et al.*, 2018) processes. In the next section, we showcase and elaborate over innovations in MEG that can further advance insight into interpersonal neural synchrony.

## Innovations in MEG analysis (canonical correlation analysis) and hardware (optically pumped magnetometers)

The approach of synchrony perception while being monitored in MEG can benefit from the implementation of advanced multivariate analysis pipelines, for instance, canonical correlation analysis (CCA). CCA is a method for finding linear relationships between two multidimensional data sets (Hotelling, 1936). The method finds signal components from the data that are maximally correlated between the datasets. Thus, CCA (or multi-set canonical correlation analysis, that is, MCCA, when extending to group contexts) naturally provides a feasible approach to study the similarity to the brain activity between subjects [for a review, see (Nicolle M Correa, Adali, *et al.*, 2010a)]. Given that social interaction and cooperation has suggested to synchronize brains during social interaction and cooperation [(Stephens *et al.*, 2010; Smirnov *et al.*, 2019); for reviews, see (Hasson *et al.*, 2012; Liu *et al.*, 2018; Nummenmaa *et al.*, 2018)], CCA may help address the question of how people infer each other’s intentions and develop mutual understanding during social interaction.

CCA has been previously applied in neuroimaging [(Stout *et al.*, 2018); for reviews, see (Nicolle M Correa, Adalı, *et al.*, 2010a); Wang et al., 2018] in experiments with simplified stimuli [e.g. (Hardoon *et al.*, 2007; Li *et al.*, 2009; Rustandi *et al.*, 2009; Nicolle M Correa, Adalı, *et al.*, 2010a; Nicolle M. Correa *et al.*, 2010b; Varoquaux *et al.*, 2010; Zhang *et al.*, 2017)], but also using more real-life stimuli, for example, observing other people in a movie [(Lankinen *et al.*, 2014; Bilenko and Gallant, 2016; Lankinen *et al.*, 2016)], experiencing multimodal naturalistic stimulation (Ylipaavalniemi *et al.*, 2009; Campi *et al.*, 2013), listening to narratives (Koskinen *et al.*, 2013; Koskinen and Seppä, 2014), using car driving simulator (Li *et al.*, 2012) or interacting with each other during MEG-hyperscanning (Remes *et al.*, 2013); Remes et al., 2015]. CCA provides a data-driven multivariate way to study mutual information between subjects’ brain responses, and thus works well, besides traditional experimental setups, also for more complex or naturalistic paradigms, such as social interaction, where building a priori model of the stimulus or interaction is difficult. CCA allows inspecting the correlation between subjects’ brain responses both in time and space: extracting the time series of the resulting signal components allows looking more closely at the temporal characteristics of synchrony, and the weights assigned to different voxels/sensors reveal the areas that contribute to the high correlations. As a multivariate method, CCA operates on the level of the whole brain in contrast to univariate methods that operate on voxel-by-voxel or sensor-by-sensor (Habeck, 2010; McIntosh & Mišić, 2013). Thus, CCA also considers spatial dependencies between brain areas that are ignored in univariate methods. This is a benefit, especially when analyzing brain activity measured during complex processes such as social interaction that can be expected to elicit activity on distributed set of brain areas. Multivariate methods also are typically less sensitive to noise and, therefore, more sensitive at a given specificity than the univariate methods (Zhuang *et al.*, 2019).

Furthermore, CCA/MCCA aligns the data from all subjects into a common space and, therefore, does not require between-subject correspondence of the sensor locations or brain areas. This is a major advantage compared to approaches where between-subject correlations are calculated directly between corresponding imaging elements or sensors [comparison for MEG in (Lankinen *et al.*, 2018)]. In the latter case, individual differences in the brain structure and function as well as between-subject variability of the head position relative to the measurement sensors typically decrease correlations. Another advantage of CCA/MCCA in the calculation of between-subject correlations of MEG/EEG data is that CCA/MCCA can be calculated at the sensor level, without the need to transform the data into the high dimensional source space. Thus, CCA/MCCA decreases the computational resources and makes the workflow simpler, as minimal preprocessing is needed. There are a number of CCA variants or extensions that can be selected with emphasis on different assumptions on the data or the paradigm [for reviews, see e.g. (Hardoon *et al.*, 2004; Klami *et al.*, 2013)]. With regard to social interaction, where the relationships of brain activities of the subjects might not be fully linear, a nonlinear version of CCA (Campi *et al.*, 2013) that measures the correlation of energies and allows for a variable delay between the time series to accommodate [see also emiCCA (Dong et al., 2015), kernel CCA and temporal kernel CCA; (Bießmann *et al.*, 2010)], for example, leader–follower changes, could be an appropriate choice. There exists a variety of advanced Bayesian CCA methods with different kind of priors or assumptions of the data that might be more effective than simpler models [Virtanen *et al.*, 2012; Klami *et al.*, 2014); for a review, see (Klami *et al.*, 2013)]. Some of them have been demonstrated to be promising in the analysis of two-person interaction in a MEG–MEG hyperscanning experiment (Remes *et al.*, 2013). Extensions of CCA by sparsity constraints [sparse CCA; (Witten and Tibshirani, 2009)] and deep learning (deep CCA; Andrew *et al.*, 2013), provide yet another possibilities for exploration of the most optimal method. In addition, there are methods that are very close to CCA/MCCA or the concept of them, such as correlated component analysis (Dmochowski *et al.*, 2012) or canonical source power correlation analysis (Dähne *et al.*, 2014). In summary, CCA has shown to be an effective tool in the analysis of similarity between multidimensional neuroimaging datasets, and promising results have been demonstrated with naturalistic and social interaction paradigms. Several extensions of CCA, allowing inspections of, for example, nonlinear relationships between the datasets, provide interesting possibilities to further investigate intersubject coupling of brain activity during social interaction as more hyperscanning studies can be expected to become available due to the development of neuroimaging techniques and the trend toward open sharing of imaging data.

Finally, beyond improvements in analysis pipelines, progress in neuroimaging infrastructures and technologies could also tap into the field of social interaction. A recent and promising technological development might enable to gradually waiver the need for indirectly measuring interactional synchrony (i.e. via synchrony perception) and ultimately achieve a very high trade-off between specificity and ecological validity. Optically pumped magnetometers (OPMs) are a state-of-the-art magnetoencephalography system that can be worn like a helmet, allowing free and natural movement (e.g. head nodding, stretching, drinking and playing a ball game) during scanning (Boto *et al.*, 2018). This emerging technology is rapidly developing, with recent data-driven modeling (Duque-Muñoz *et al.*, 2019) and improved detection of the movement-sensitive gamma-band activity (Iivanainen *et al.*, 2019), thus advancing forwards as a viable, mobile alternative to traditional and static MEG neuroimaging. This new neuroimaging venue is very promising for the study of interactional synchrony and is most probably holds the highest potential for obtaining high neuronal specificity during real-life settings. Of course any new technology requires further refinement to be fully and optimally exploited, and in the coming years, social scientists should test and experiment with this new neuroimaging tool, in dyadic, as well as group social settings. As this development continues to advance, it would open new possibilities for scanning social interactions with spatial and temporal acuity thus far impossible to obtain.

## Summary

There is a growing trend among neuroscientists to adapt realistic experimental settings, so-called ‘in the wild’, in their research. Integrating the integration of behavioral and neural synchrony (i.e. biobehavioral synchrony) into this trend and using robust methodology to study the complex and diverse aspects of social life is timely and promising for generating exciting new applications. We present here several methodological innovations that have the potential to advance the field of interactive neural synchrony. We begin by delineating the brief, but rapid progress in the neuroscientific study of social interaction within less than a decade we have witnessed a shift from applying static artificial stimulation of interaction, to real-life interaction in the wild. We elaborated on the importance of integrating interactive neural synchrony with various measures of nonverbal behavior, in order to capture and explain complex societal phenomena. We also discussed the major methodological challenge that this field is facing: the trade-off between ecological validity and experimental rigor. We then highlight the use of MEG neuroimaging as a promising approach for mitigating this trade-off and increasing the richness and quality of neural data collected during real-life social interactions. We end with presenting methodological innovations that have the potential to further advance this field: data-driven multivariate analysis (CCA) and mobile MEG (OPM). These are exciting times for conducting research into the intrinsic mechanisms that drive social life and for the application of novel computational methods, new experimental contexts and new real-life experimental paradigms.

## Funding

The work was supported by the Academy of Finland Research Fellow funding and a NARSAD Young Investigator Grant from the Brain & Behavior Research Foundation to J.L., and by grants to R.F. from the Irving B. Harris Foundation and the Simms/Mann Foundations.



*Conflict of interest*: None declared.
